# Genomics and epigenomics

**DOI:** 10.1186/1129-2377-16-S1-A7

**Published:** 2015-09-28

**Authors:** Paolo Garagnani, Rossana Terlizzi, Sabina Cevoli, Sabina Capellari, Giulia Pierangeli, Chiara Pirazzini, Maria Giulia Bacalini, Claudio Franceschi, Pietro Cortelli

**Affiliations:** Dipartimento di Medicina Specialistica Diagnostica e Sperimentale, Università di Bologna, Bologna, Italy; IRCCS Istituto delle Scienze Neurologiche, Bologna, Italy; Dipartimento di Scienze Neurologiche, Università di Bologna, Bologna, Italy

Migraine with (MA) and without aura (MO) is a common brain disorder that affects 15% of the general population. Genetic studies on twins have shown that MA and MO heritability spans between 50% and 60%[1]. Despite the high degree of heritability the genetic basis of MA and MO has not been elucidated and on the whole their etiology is far from being resolved. Several years ago it has been hypothesized that also epigenetic mechanisms such as DNA methylation, miRNA and histone modifications could play a relevant role in MA and MO. In particular epigenetic mechanisms have the potential to link early life events, neuro-inflammation and estrogen activities in the etiology of migraine and in its chronification[2] and pharmaco-epigenetics could be implicated in the wide spectra of different drug treatment responses[3].

In recent years the technologies for studying nucleic acids have literally exploded, opening to new possibilities for study of genetics and epigenetics of MA and MO[4].

One of the most significant results is the sharp cost decrease for the whole genome DNA sequencing, since the psychological threshold of 1000$ for a 30X genome is about to be achieved. This cost reduction is fostering a wealth of large sequencing campaigns that will allow overcoming all the limitations due to the poor knowledge of human genetic variability that has plagued the ability of identifying the genetic basis of all sporadic diseases including MA and MO[5].

The reduction of nucleic acids sequencing costs and the availability of cost effective microarray solutions for the analysis of DNA methylation has favored the implementation of epigenomic studies, in particular DNA methylation microarray has been thoroughly used providing new insight regarding the variability and the role of such epigenetic agent. DNA methylation, miRNA and histone modifications have proven to be a potential source of powerful and robust biomarkers.

Taken together both the new genetic and epigenetic omic approaches have the potential to provide new molecular insight in the etiology of MA and MO. Moreover from such approaches we expect to obtain tools to improve migraine diagnosis, patient stratification, and therapy.

## Conflict of interest

None.
